# The impact of COVID-19 and national pandemic responses on health service utilisation in seven low- and middle-income countries

**DOI:** 10.1080/16549716.2023.2178604

**Published:** 2023-03-07

**Authors:** Donald Fejfar, Afom T. Andom, Meba Msuya, Marc Antoine Jeune, Wesler Lambert, Prince F. Varney, Moses Banda Aron, Emilia Connolly, Ameyalli Juárez, Zeus Aranda, Anne Niyigena, Vincent K. Cubaka, Foday Boima, Vicky Reed, Michael R. Law, Karen A. Grépin, Jean Claude Mugunga, Bethany Hedt-Gauthier, Isabel Fulcher

**Affiliations:** a Clinical, Partners In Health, Boston, MA, USA; b Clinical Services, Partners In Health, Maseru, Lesotho; c Department of Strategic Planning and Information Systems, Zanmi Lasante, Croix-des-Bouquets, Haiti; d Strategic Health Information Systems, Partners In Health, Monrovia, Liberia; e Monitoring, Evaluation, and Information, Partners In Health, Neno, Malawi; f Partners In Health/Compañeros en Salud, Jaltenango de la Paz, Mexico; g Department of Research and Training, Partners In Health, Kigali, Rwanda; h Strategic Health Informations Systems, Partners In Health, Koidu City, Kono District, Sierra Leone; i Centre for Health Services and Policy Research, School of Population and Public Health, University of British Columbia, Vancouver, BC, Canada; j School of Public Health, Li Ka Shing Faculty of Medicine, The University of Hong Kong, Pokfulam, Hong Kong; k Department of Global Health and Social Medicine, Harvard Medical School, Boston, MA, USA

**Keywords:** Global public health, Covid-19, essential health services, time series modeling, health policy

## Abstract

**Background:**

The COVID-19 pandemic has disrupted health services worldwide, which may have led to increased mortality and secondary disease outbreaks. Disruptions vary by patient population, geographic area, and service. While many reasons have been put forward to explain disruptions, few studies have empirically investigated their causes.

**Objective:**

We quantify disruptions to outpatient services, facility-based deliveries, and family planning in seven low- and middle-income countries during the COVID-19 pandemic and quantify relationships between disruptions and the intensity of national pandemic responses.

**Methods:**

We leveraged routine data from 104 Partners In Health-supported facilities from January 2016 to December 2021. We first quantified COVID-19-related disruptions in each country by month using negative binomial time series models. We then modelled the relationship between disruptions and the intensity of national pandemic responses, as measured by the stringency index from the Oxford COVID-19 Government Response Tracker.

**Results:**

For all the studied countries, we observed at least one month with a significant decline in outpatient visits during the COVID-19 pandemic. We also observed significant cumulative drops in outpatient visits across all months in Lesotho, Liberia, Malawi, Rwanda, and Sierra Leone. A significant cumulative decrease in facility-based deliveries was observed in Haiti, Lesotho, Mexico, and Sierra Leone. No country had significant cumulative drops in family planning visits. For a 10-unit increase in the average monthly stringency index, the proportion deviation in monthly facility outpatient visits compared to expected fell by 3.9% (95% CI: −5.1%, −1.6%). No relationship between stringency of pandemic responses and utilisation was observed for facility-based deliveries or family planning.

**Conclusions:**

Context-specific strategies show the ability of health systems to sustain essential health services during the pandemic. The link between pandemic responses and healthcare utilisation can inform purposeful strategies to ensure communities have access to care and provide lessons for promoting the utilisation of health services elsewhere.

## Introduction

The COVID-19 pandemic has profoundly disrupted the utilisation of essential health services worldwide, potentially contributing to worsened mortality rates, secondary disease outbreaks, and the exacerbation of existing health inequities [1–6]. Leading health organisations, such as the World Health Organization (WHO), have warned that severe disruptions to health systems due to the pandemic can lead to interruptions in the continuity of health services [7]. Despite these warnings, 92% of 129 countries surveyed by the WHO continued to report disruptions to essential health services a year and a half into the pandemic [8].

The degree of disruption has varied by type of health service, patient population, and geographic area [5,9,10]. A systematic review in August 2020 of 81 studies across 20 countries found that outpatient visits at health facilities were the service most impacted by the COVID-19 pandemic [11]. Greater reductions in utilisation were observed in people with a milder spectrum of illness, indicating that individuals with more serious illness may have continued to seek care [11]. Comparatively, maternal health services have been minimally affected in some settings, indicating continued care for some planned and necessary services [5,8,12–15]. While the magnitude of disruptions has varied greatly by geography and service type, high-income countries generally reported fewer services disrupted than low- and middle-income countries (LMICs) [8].

Explanations for why disruptions in essential health services occur involve a combination of drivers, including infection mitigation strategies such as lockdowns [5,9,16–18], patient fear of contracting COVID-19 [16,19–21], and supply constraints due to triaging health system resources amidst COVID-19 waves [8]. However, isolating the precise reason for any single given disruption has been challenging for two reasons. First, factors contributing to disruptions are often interrelated; countries rarely implement isolated measures and they are also not implemented randomly, but rather at peak times [7,8,22]. For example, increasing COVID-19 case counts may trigger government-imposed lockdowns, which may both restrict a patient’s mobility and affect their willingness to seek care. Second, the intensity of ‘lockdowns’ and other restrictions are difficult to precisely define and measure as they vary widely in their design and implementation [23].

The stringency index developed by the Oxford COVID-19 Government Response Tracker provides a standardised measurement for the intensity of national pandemic containment responses across all countries in the world. Of the few studies that have examined the relationship between health service utilisation and the stringency index, all have done so on a national level and the majority have only modelled changes in outpatient visits [5,9]. We have two main goals in our study. First, recognising the potential of differential impact to utilisation by service type, we investigate the impact of the COVID-19 pandemic on three key essential services – outpatient visits, facility-based deliveries, and family planning services – in seven LMICs: Haiti, Lesotho, Liberia, Malawi, Mexico, Rwanda, and Sierra Leone. Secondly, we utilise the Oxford stringency index to quantify the relationship between the intensity of national responses and disruptions to local facility-level health service utilisation. We focus on a long-term and local perspective, reporting cumulative effects on health services with context from local staff.

## Methods

Partners In Health is a global non-government organisation that strengthens health systems by supporting the public facility operations of health ministries. This study included routinely collected data from Partners In Health sites in the seven aforementioned LMICs serving a total of 16 district-level areas and 104 facilities (Table 1). All of the facilities included are in rural locations, and details on the major programme foci in each included country can be found at pih.org/countries.

**Table 1.** National- and district-level features of countries in the study on health service utilisation during the COVID-19 pandemic, 2020–2021.

Feature	Haiti	Lesotho	Liberia	Malawi	Mexico	Rwanda	Sierra Leone
**Overview of participating Partners In Health locations**							
Local organization name	Zanmi Lasante	Bo-mphato Litsebeletsong Tsa Bophelo	Partners In Health Liberia	Abewenzi Pa Za Umoyo	Compañeros En Salud	Inshuti Mu Buzima	Partners In Health Sierra Leone
Supported districts included in this study	Central plateau and lower Artibonite	Mohale’s Hoek, Thaba Tseka, Mokhotlong, and Qacha’s Nek	Harper, Pleebo, and Karluway 1	Neno	Sierra and Fraylesca Regions, Chiapas	Burera, Kayonza, and Kirehe	Kono
# health facilities reported on	14	7	4	15	12	50	2
Health services data source	Internal PIH database	Internal PIH database	DHIS-2	DHIS-2	OpenMRS EMR (outpatient visits); Excel spreadsheets (facility-based deliveries and family planning)	DHIS-2	Internal PIH database
**National COVID-19 situation through December 31, 2021**							
First COVID case, 2020 [24]	March	May	March	April	March	March	March
Cumulative COVID-19 cases, per million people [25]	2,251	13,734	1,212	3,859	30,622	8,485	874
Cumulative case fatality rate, % [26]	2.95	2.26	4.57	3.12	7.51	1.20	1.73

PIH: Partners In Health; DHIS-2: District Health Information System-2.

We selected three indicators to represent health service utilisation across our sites: outpatient visits, facility-based deliveries, and family planning services. These indicators were chosen because they were widely available in routine health data systems, have high-quality data, and represent different types of care: urgent, unplanned care with outpatient visits; urgent, semi-planned care with facility-based deliveries; and non-urgent semi-planned care with family planning services. Details of indicator definitions by site are provided in the supplementary material (Supplement 1). Indicator data included monthly counts aggregated at the facility-level.

We used the ‘stringency index’ from the Oxford COVID-19 Government Response Tracker as a measure of the intensity of national-level containment strategies. The stringency index is made up of nine individual components, including school and workplace closures, cancellations of public events, restrictions on gathering sizes, closures of public transport, stay-at-home requirements, restrictions on internal movement and international travel, and public information campaigns. We chose the stringency index for this work because it is rigorously and regularly collected, publicly available and standardised across all of our sites, and includes multiple components of restrictions important to service access and the reasons why changes in healthcare utilisation may occur. These components are aggregated into a single ‘index’: a number ranging from 0–100 [27]. We pooled daily reports to compute a monthly average of this index for each country to match our monthly-level data.

For our health service outcomes, we first computed deviations in health service utilisation from March 2020 through December 2021 for each indicator separately for each country. We modelled monthly counts at the facility level based on yearly trends and seasonality using baseline data from January 2016 to February 2020 using a negative binomial time series model. We used these models to extrapolate predicted counts for each indicator from March 2020 to December 2021. Predicted counts were aggregated across facilities and compared to their observed values to compute country-level monthly deviations from expected with 95% prediction intervals for each indicator. We report estimated cumulative deviations reported as counts and proportion deviations for each indicator and country. Additional detail on our statistical modelling approach is detailed in Fulcher et al. [28].

After the results were available, analysts and co-authors conducted meetings with 1–3 co-authors from each respective PIH site, in addition to any invited site-specific research staff and clinicians, to interpret the modelling results. Feedback was also obtained during three one-hour meetings with the Partners In Health Cross-site COVID-19 Working Group, a team of researchers and clinicians from Harvard Medical School and PIH that meets regularly to discuss COVID-19 research at PIH-supported sites. These meetings were held to ensure that important stakeholders familiar with the data, clinical settings, and local policies were able to provide context qualitatively.

In most countries, data for family planning and facility-based deliveries were available at all PIH-supported facilities included. However, in Mexico, data on facility-based deliveries and family planning were only available at a single community hospital and adjacent birthing centre and in Malawi, data were not available for five facilities without maternity wards. Facilities were excluded for having: less than 80% baseline data, any missing data during the pandemic era, or a median monthly count of zero during the pandemic era. In total, this resulted in 26 of 276 (9.4%) of facility-indicator combinations being excluded from analyses, including both facilities in Sierra Leone for family planning visits. Residual plots were checked for all time-series models, and all models were assessed for autocorrelation (with no adjustments necessary).

Second, for each indicator, we investigated the relationship between the monthly average stringency index and monthly proportion deviations in utilisation. We operationalised deviations in two ways: (1) estimated proportion deviation, defined as the deviation in observed cases from expected divided by the number of expected cases and (2) a binary indicator marking months with a deviation significantly lower than expected (i.e. the observed value fell below the 95% prediction interval). For each indicator, we estimated the (1) mean change in proportion deviation with linear regression and (2) the log odds of significantly lower deviation than expected with logistic regression, reporting one marginal effect estimate aggregating across all countries. Models accounted for the country via random intercept terms. We also adjusted for the number of COVID-19 cases per capita for the prior month; the number of COVID-19 cases circulating may be a reason for reduced patient care-seeking behaviour due to a fear of contracting COVID-19 [16,19–21]. For any model where the p-value of the average monthly stringency index was significant at the α = 0.05 significance level, we also fit an additional linear regression model for each component making up the stringency index, controlling for the same terms described above (lagged COVID-19 cases and country random effects).

All data cleaning, statistical analyses, and data visualisations were produced in R V4.1.0. Random effects models were fit and marginal effect estimates were calculated using the GLMMadaptive package.

## Results

**Figure 1.**
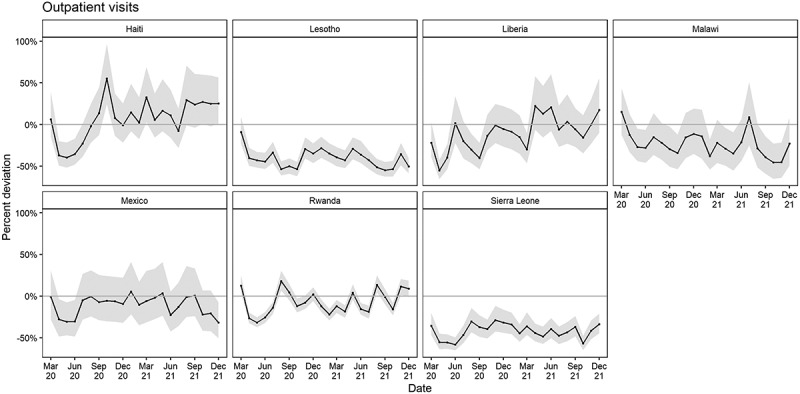
Estimated percent deviation in outpatient visits (difference in observed count from expected in black with 95% prediction interval in grey), by month and country, March 2020 – December 2021.

From March 2020 to December 2021, the estimated cumulative percent difference in outpatient visits was 1.4% (−10.3%, 8.7%) less than expected in Haiti; 38.9% (−43.2, 35.0) less than expected in Lesotho; 18.6% (−27.7%, −10.2%) less than expected in Liberia; 21.6% (−31.6%, −11.8%) less than expected in Malawi; 11.2% (−20.5%, 0.0%) less than expected in Mexico; 11.8% (−15.0%, −8.9%) less than expected in Rwanda; and 41.5% (−46.2%, −37.1%) less than expected in Sierra Leone (Table 2). A significant cumulative deviation in outpatient visits during the pandemic occurred in Lesotho, Liberia, Malawi, Rwanda, and Sierra Leone (Figure 1).

**Table 2.** Cumulative deviation in health service utilisation from baseline (January 2016 – February 2020) during the study period (March 2020 – December 2021).

Country, indicator	Cumulative observed counts (true value)	Cumulative deviation from expected counts (95% Prediction Interval)	Cumulative percent deviation (95% Prediction Interval)
**Haiti**			
Outpatient visits	1,539,840	−13,198.0 (−107,119.0, 75061.1)	−1.4% (−10.3%, 8.7%)
Deliveries in health facilities	38,216	−4,472.0 (−6,209.7, −3,012.5)	−16.6% (−21.6%, −11.8%)
Family planning services	13,653	436.5 (−1,204.1, 1,795.0)	5.3% (−12.1%, 26.0%)
**Lesotho**			
Outpatient visits	60,837	−25,086.5 (−30,016.9, −21,240.7)	−38.9% (−43.2%, −35.0%)
Deliveries in health facilities	1,509	−126.0 (−257.5, −10.0)	−11.4% (−20.9%, −1.0%)
Family planning services	17,427	−782.0 (−2,145.7, 348.4)	−6.3% (−15.5%, 3.1%)
**Liberia**			
Outpatient visits	131,538	−17,641.5 (−29,557.4, −8,792.4)	−18.6% (−27.7%, −10.2%)
Deliveries in health facilities	3,306	−187.0 (−441.1, 14.0)	−8.7% (−18.3%, 0.7%)
Family planning services	37,754	−6,776.5 (−15,274.3, 303.3)	−21.5% (−38.1%, 1.2%)
**Malawi**			
Outpatient visits	530,054	−95,520.0 (−159,718.9, −46,299.8)	−21.6% (−31.6%, −11.8%)
Deliveries in health facilities	8,912	−86.5 (−458.6, 240.2)	−1.5% (−7.4%, 4.4%)
Family planning services	2,435	245.5 (−99.3, 493.0)	19.2% (−6.1%, 47.8%)
**Mexico**			
Outpatient visits	31,019	−2,544.0 (−5,210.6, 9.4)	−11.2% (−20.5%, 0.0%)
Deliveries in health facilities	1,095	−116.5 (−218.0, −21.0)	−14.7% (−24.3%, −3.0%)
Family planning services	1,151	−140.0 (−442.5, 55.6)	−17.9% (−40.8%, 9.5%)
**Rwanda**			
Outpatient visits	1,973,750	−167,037.5 (−219,404.4, −121,458.3)	−11.8% (−15.0%, −8.9%)
Deliveries in health facilities	45,205	−746.0 (−1,553.6, 28.1)	−2.6% (−5.3%, 0.1%)
Family planning services	2,897,781	−3,191.5 (−46,417.2, 39083.4)	−0.2% (−2.4%, 2.1%)
**Sierra Leone**			
Outpatient visits	117,982	−50,380.0 (−60,915.5, −41,793.1)	−41.5% (−46.2%, −37.1%)
Deliveries in health facilities	4,610	−504.0 (−748.6, −301.8)	−15.7% (−21.6%, −10.0%)
Family planning services	NA	NA	NA

**Figure 2.**
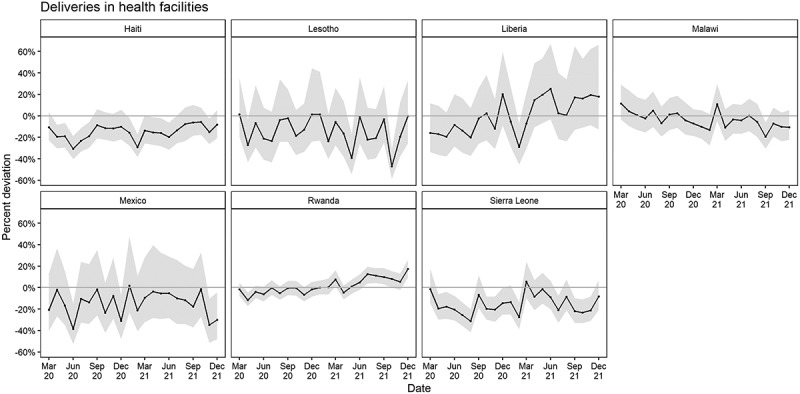
Estimated percent deviation in deliveries in health facilities (difference in observed count from expected in black with 95% prediction interval in grey), by month and country, March 2020 – December 2021.

The estimated cumulative percent difference in facility-based deliveries was 16.6% (−21.6%, −11.8%) less than expected in Haiti; 11.4% (−20.9%, −1.0%) less than expected in Lesotho; 8.7% (−18.3%, 0.7%) less than expected in Liberia; 1.5% (−7.4%, 4.4%) less than expected in Malawi; 14.7% (−24.3%, −3.0%) less than expected in Mexico; 2.6% (−5.3%, 0.1%) less than expected in Rwanda; and 15.7% (−21.6%, −10.0%) less than expected in Sierra Leone (Table 2). A significant cumulative deviation in facility-based deliveries from March 2020 to December 2021 was observed in Haiti, Lesotho, Mexico, and Sierra Leone (Figure 2).

**Figure 3.**
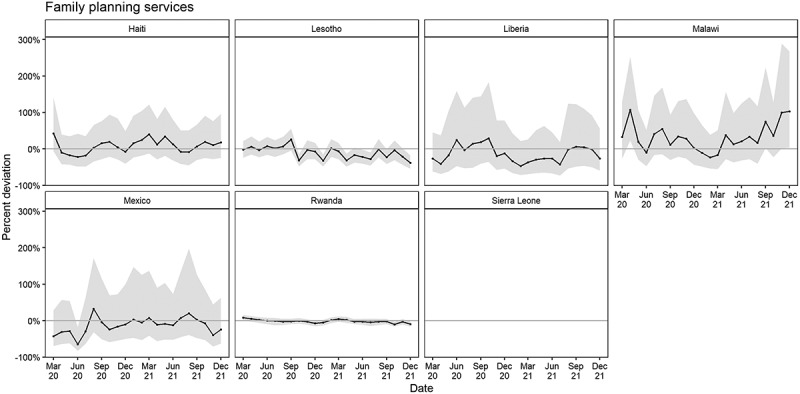
Estimated percent deviation in family planning services (difference in observed count from expected in black with 95% prediction interval in grey), by month and country, March 2020–December 2021.

The estimated cumulative percent difference in family planning services was 5.3% (−12.1%, 26.0%) more than expected in Haiti; 6.3% (−15.5%, 3.1%) less than expected in Lesotho; 21.5% (−38.1%, 1.2%) less than expected in Liberia; 19.2% (−6.1%, 47.8%) more than expected in Malawi; 17.9% (−40.8%, 9.5%) less than expected in Mexico; and 0.2% (−2.4%, 2.1%) less than expected in Rwanda. Both facilities in Sierra Leone were excluded for missing baseline data (Table 2). A significant cumulative deviation in family planning services from March 2020 to December 2021 was not observed for any country (Figure 3).

*Both facilities in Sierra Leone were excluded for family planning (see Methods)

The average stringency index varied greatly by country and month (Supplement 3). The lowest average stringency index across all months was in Sierra Leone (41.4) and the highest was in Mexico (66.6). In all countries, the stringency index varied the most in March 2020 when restriction measures began. The correlation between components of the stringency index ranged from 0.02 to 0.61 (between internal movement controls and school closings) (Supplement 5).

For both the linear and logistic regression models, only outpatient visits had a significant association (*p* < 0.05) with the stringency index (Table 3). For a 10-unit increase in the average monthly stringency index, the proportion deviation in outpatient visits dropped by an average of 3.9% (95% CI: −5.1%, −1.6%). Further, the odds of a deviation significantly lower than expected are 1.60 (95% CI: 1.15, 2.24) times higher for a 10-unit increase in the average monthly stringency index. Full model results are given in the supplementary material (Supplement 4).

**Table 3.** Relationship between the average monthly stringency index (fixed effect) and estimated deviation in health service utilisation, adjusted for country (random effect) and 1-month lagged monthly COVID-19 cases per 1,000 people (fixed effect).

Outcome	Regression type	Indicator	Stringency index beta coefficient (95% CI)	*p*-value
Estimated proportion deviation	Linear	Outpatient visits	−0.0039 (−0.0051, −0.0016)	0.0002*
		Deliveries in health facilities	−0.0011 (−0.0023, 0.0004)	0.0578
		Family planning services	−0.0005 (−0.0028, 0.0026)	0.9721
Binary indicator for a significant decrease in utilization (below 95% PI)	Logistic	Outpatient visits	0.0472 (0.0137, 0.0807)	0.0057*
		Deliveries in health facilities	−0.0012 (−0.0275, 0.0251)	0.9288
		Family planning services	−0.0272 (−0.0751, 0.0207)	0.2652

*Indicates a p-value below the 0.05 significance-level cutoff.

For outpatient visits, we fit separate linear regression models to investigate the relationship between monthly deviation and each component in the stringency index. We found that school closings, workplace closings, public transport closings, stay at home requirements, and restrictions on internal movement were all significantly associated with a decrease in outpatient visits (Supplement 6). However, public event cancellations, restrictions on gatherings, international movement controls, and public information campaigns were not associated with changes in outpatient visit attendance.

## Discussion

All countries included in our study experienced significant sudden and cumulative drops in outpatient visits during the COVID-19 pandemic. Numerous research studies and global surveys corroborate these findings with significant declines in outpatient visits and general clinic attendance observed throughout the COVID-19 pandemic [5,7–11,13,22]. We further found that disruptions to outpatient visits were related to increased stringency of national responses. Specifically, outpatient visits at PIH-supported sites in Haiti, Liberia, Mexico, and Rwanda rebounded towards normal after the first three months of the pandemic when pandemic restrictions loosened, as measured by the stringency index (Supplement 3). This is similar to findings from other studies quantitatively observing the relationship between lockdowns or similar measures and levels of outpatient visits, including three multi-country studies with data from Haiti, Mexico, Liberia, Malawi, and Sierra Leone [5,9,10,29].

Although it is difficult to parse out reasons for disruptions to services, PIH site teams reported a number of reasons for the declines and fluctuations observed locally in outpatient visits. For example, many of our staff (especially in Haiti, Liberia, and Malawi) reported drops in cases due to more serious lockdowns and fear of contracting COVID-19. This is especially true for disruptions early in the pandemic which occurred in nearly every country. Staff in Haiti, Mexico, Rwanda, and Liberia also noted drops corresponded to when COVID case counts were highest and lockdowns (and thus the stringency index) were stricter. PIH sites in Sierra Leone actively recruited patients via community outreach to return to care for health maintenance and chronic visits when allowed. Drops in cases at PIH sites were also notably related to the rurality and accessibility of sites, such as availability of transport during lockdowns. In Lesotho, the largest drops were observed in Lebakeng and Manamaneng, which are the hardest to reach. In Mexico, despite fear for seeking care reported by providers, closures of other clinics meant that PIH hospitals were the only places to go for care in these areas, keeping cases closer to normal. Staff also noted specific strategies undertaken to keep hospitals open to needy patients, such as giving patients 3 months’ worth of medications (rather than 1); these chronic care patients, as well as pregnant patients, were asked to space out their visits more than normal. In fact, one of the major drops observed in Mexico can be attributed to a partial local forced closure by citizens and providers in November and December 2021. Finally, in Haiti, rebounds were reported starting October 2020 that could be due to the loosening of COVID restrictions (especially regarding healthcare visits) and out of district patients being referred to PIH hospitals and clinics. Significant cumulative decreases in the number of facility-based deliveries were observed in Haiti, Lesotho, Mexico, and Sierra Leone. In all countries but Mexico and Haiti, facility-based deliveries were less affected by COVID-19 than outpatient visits, although there were still significant drops at several points throughout the pandemic. Our mixed findings are corroborated in the literature, which showed sporadic reductions during the pandemic and variation by geographic locale [11,30,31]. Unlike outpatient visits, disruptions in facility-based deliveries were not significantly correlated with the intensity of national pandemic responses. In Kinshasa, DRC, Hategaka et al. [29] observed no impact of the pandemic or lockdowns on facility-based childbirth, similar to our findings. In the one multi-country study reporting on this relationship, weak correlations were observed for maternal and child services compared to services such as outpatient visits, similar to the results found in our study for facility-based deliveries in comparison to outpatient visits [9].

Based on feedback from meetings with PIH site teams, facility-based births were maintained despite pandemic restrictions. For example, the presence of maternal waiting homes that operated despite other COVID-19 restrictions in Lesotho provided safety and ease of transportation for mothers to attend facilities for deliveries, which may explain why facility-based deliveries remained as expected compared to drops in outpatient visits. In Rwanda, we observed below-expected levels of facility-based deliveries in April 2020, similar to the 10% drops in March and April 2020 observed by Wanyana et al. nationally [32]. Exceptions to some national pandemic-related restrictions on movement in Rwanda were given to pregnant mothers to allow them to attend health facilities for delivery, which may account for some of the return to normal levels as the pandemic progressed that we observed in our study. In other countries without specific strategies to maintain visits during the pandemic, staff often cited the importance of maternal health programmes at PIH supported sites as reasons for the maintenance of care, especially after the initial part of the pandemic when minor drops could be seen in certain months.

Access to family planning services was the least affected by the COVID-19 pandemic with no significant cumulative reductions in any country and no correlation with the intensity of national response. Site staff discussed a number of explanations: this may have been due to continuity of family planning services provided by community health workers, community dialogue, and promotion of these services by facilities in PIH-supported catchment areas. Although representing a different type of care, this shows a similarity to facility-based deliveries as a highly important focus area for PIH supported sites in all of the included countries. For example, in Malawi and Sierra Leone, a large programme began in August 2019 supporting community and clinical facility-based outreach arms and advocacy for family planning services [33]. At PIH-supported sites in Mexico, receipt of birth control methods does not require proof of residency or provider prescription; this ease of access may have sustained pre-COVID-19 levels of family planning service utilisation. Indeed, barriers to accessing family planning have been cited as reasons for reductions in utilisation of family planning during COVID-19 in Kenya and Ethiopia [34,35]. However, family planning services, such as the provision of in-demand birth control methods, are often administered after facility-based delivery, which may explain the slight decrease in the first few months of the pandemic paralleling the trend with facility-based deliveries. In Rwanda, family planning is similarly administered immediately after deliveries, which may explain the maintenance of family planning alongside sustained levels of facility-based deliveries [36].

Overall, we found that service types were differentially impacted by the stringency of national pandemic responses. These results are similar to the few other studies measuring this relationship, with the one study quantifying this relationship disaggregated by service type reporting varying correlations depending on service type [5,9,10]. The WHO released results from ‘Pulse Surveys’ on essential health services, which noted that a mix of supply-side factors (e.g. resourcing of health systems) and demand-side factors (e.g. care seeking) is responsible for disruptions, which are highly dependent on service type and region [7,8,22]. Notably, the WHO found no correlation between the Oxford stringency index and overall service disruption by country. This aggregated analysis may have masked possible associations with specific health services. However, our analysis of disruptions in outpatient visits in relation to the individual components of the stringency index found significant associations with factors that repeatedly came up in discussions with site staff about why disruptions may have occurred, including school and workplace closings, stay at home requirements, and reductions in access to public transport due to closures. These may be especially important due to the rurality of many of the study sites.

The results of this study may be limited by several factors. First, we estimated a single effect measure for the stringency index aggregated across all included countries. If the relationship between stringency index and utilisation varied by country, we would not be able to detect this in our analysis. Second, our study used data aggregated at the monthly and facility level, which precluded disaggregation by potentially important patient characteristics such as age or gender. These indicators also vary slightly in definition country to country; our ability to closely compare results across countries and generalise more broadly may also be slightly limited by this fact. Third, number of outpatient visits is a catchall indicator that represents a broad variety of services, and we were unable to investigate if the COVID-19 pandemic had greater impact on certain types of outpatient services (i.e. differences in chronic care visits versus acute outpatient visits). Fourth, the stringency index is a national measure, and thus may not be applicable to the PIH-supported sites, which represent remote and vulnerable sub-national areas. These areas may experience differences typical of more rural areas, such as a lower stringency of restriction measures than at the national level (e.g. non-adherence to national lockdowns). Further, our analysis also used national-level COVID-19 cases as a proxy to account for patient fear of seeking care during the pandemic, which suffers from similar drawbacks as the stringency index and is also subject to underreporting during the months of study [37,38]. PIH-supported sites may also differ from other facilities in health service utilisation.

## Conclusions

We observed declines in outpatient visits across all countries which were associated with the stringency of national pandemic responses. The impact of the pandemic on facility-based deliveries and family planning services varied over time and across countries. Facility-based deliveries (a service that cannot be delayed) and family planning (a service frequently relying on community outreach) were both largely maintained at our study sites despite national pandemic responses. Understanding the reasons why these services were sustained while outpatient visits decreased during the pandemic provides lessons for how to maintain essential health services during prolonged health system emergencies. Future research should investigate the long-term impact of these prolonged disruptions on morbidity and mortality.

## Supplementary Material

Supplemental Material

## Data Availability

Data from this study may be shared upon reasonable request to the corresponding author and approval from country-teams.

## Paper context

COVID-19-related disruptions to essential health services have been documented worldwide. However, few studies assess the relationship between the intensity of national pandemic containment responses and their impact on essential health service utilisation during the COVID-19 pandemic. This study quantitatively links the intensity of national-level pandemic containment responses to the utilisation of outpatient services. We also provide information on the mitigation strategies employed by specific countries to maintain essential health services during the COVID-19 pandemic.
