# Supplementary material for: The impact of COVID-19 and national pandemic responses on health service utilisation in seven low- and middle-income countries
Source: Glob Health Action. 2023 Mar 7;16(1):2178604. doi: 10.1080/16549716.2023.2178604 (PMC10013493; doi:10.1080/16549716.2023.2178604)
Supplement: Supplemental Material [file ZGHA_A_2178604_SM1164.docx]

# Supplementary Material

**S1.** Site-specific definitions of indicators included in the study on health service utilization during the COVID-19 pandemic, 2020-2021

| Country | Outpatient visits | Deliveries in health facilities | Family planning services |
| --- | --- | --- | --- |
| Haiti | Total number of outpatient attendances or primary care visits | Number of facility-based births | Number of women and girls receiving (a) oral and (b) injectable contraceptives |
| Lesotho | Visits consulted at the outpatient department in facilities Excludes patients for ANC, deliveries, PNC, TB, ART, pediatrics (under 5), HIV testing, and other clients not seen for acute or chronic illness | Deliveries at health facilities | Headcount of patients who received family planning commodities |
| Liberia | All outpatient visits | Total deliveries (combined count of deliveries by skilled and unskilled birth attendants and assisted deliveries) | Total number of family planning methods given (new and continued users) across a variety of types |
| Malawi | Visits consulted at the outpatient department. Excludes patients for ANC, PNC, TB, ART, HIV testing, immunizations, and other clients considered inpatient or another visit type | All deliveries conducted at 10 health facilities. Five facilities excluded which do not have maternity wards | Total number of family planning methods given (new and continued users) across a variety of family planning methods |
| Mexico | All outpatient visits | Deliveries at health facilities with maternity wards (one birthing center and one other facility) | Number of family planning methods given at facilities with maternity wards: includes monthly, bimonthly, and quarterly injections, emergency and non-emergency oral contraceptive pills, implants, IUDs, condoms, and bilateral tubal occlusion. Every unit is one type of method delivered in counting |
| Rwanda | All patients seen in OPD wards (district hospital or health center) and patients seen in all other special services such as NCD, HIV, ID, etc. (both new and existing cases) | Deliveries are counted as total numbers of women delivered at a health facility | Number of family planning methods given during each month (new and long-term users). This also included condoms provided at a health facility |
| Sierra Leone | Outpatient department visits of types: adult, pediatric (under 5), and ANC | Number of facility-based deliveries | Headcount of patients receiving family planning services at the facility |

**S2.** Time series analyses of selected indicators pre-/post-COVID-19 pandemic (dotted line at March 2020), observed count (black) and estimated count with 95% prediction interval (purple)


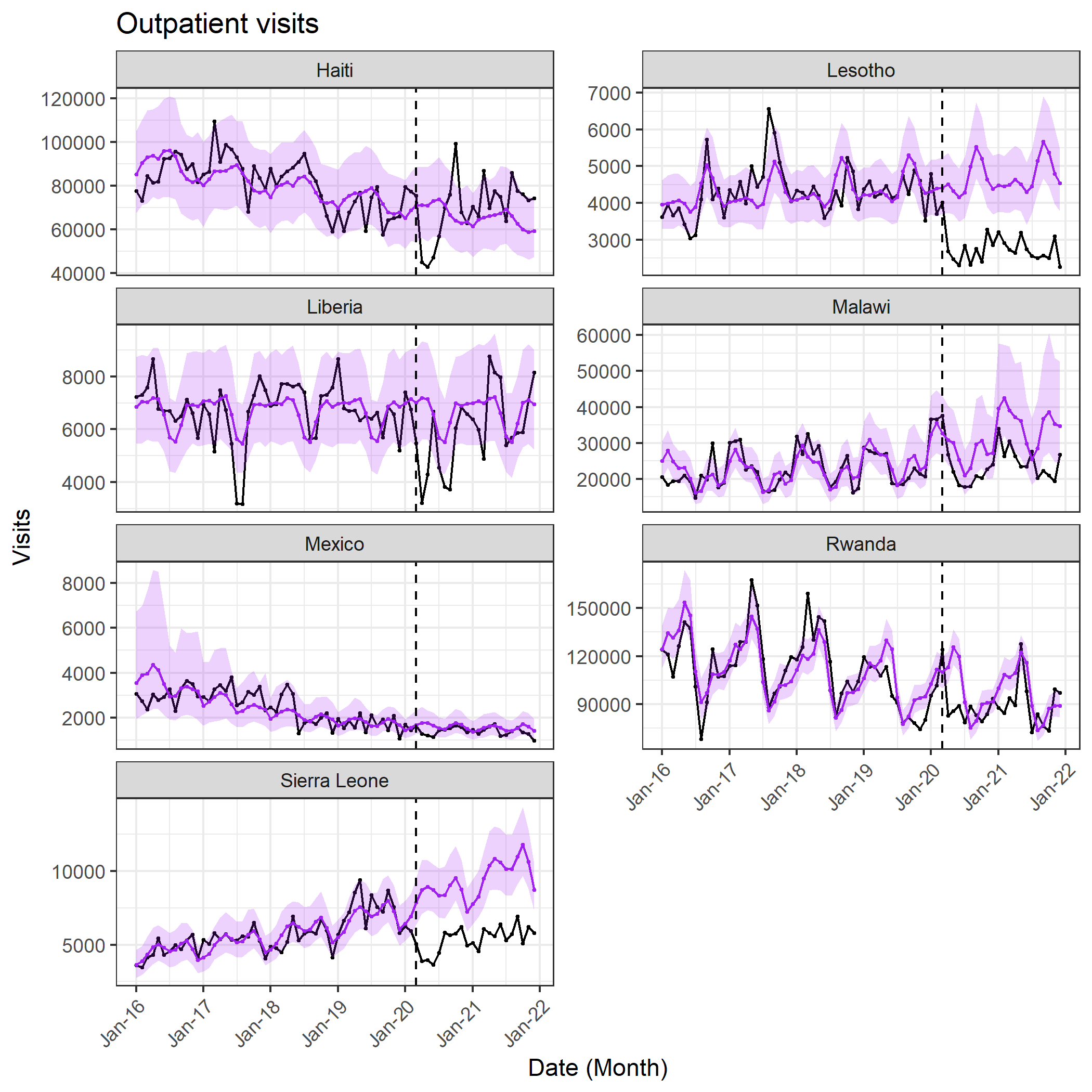

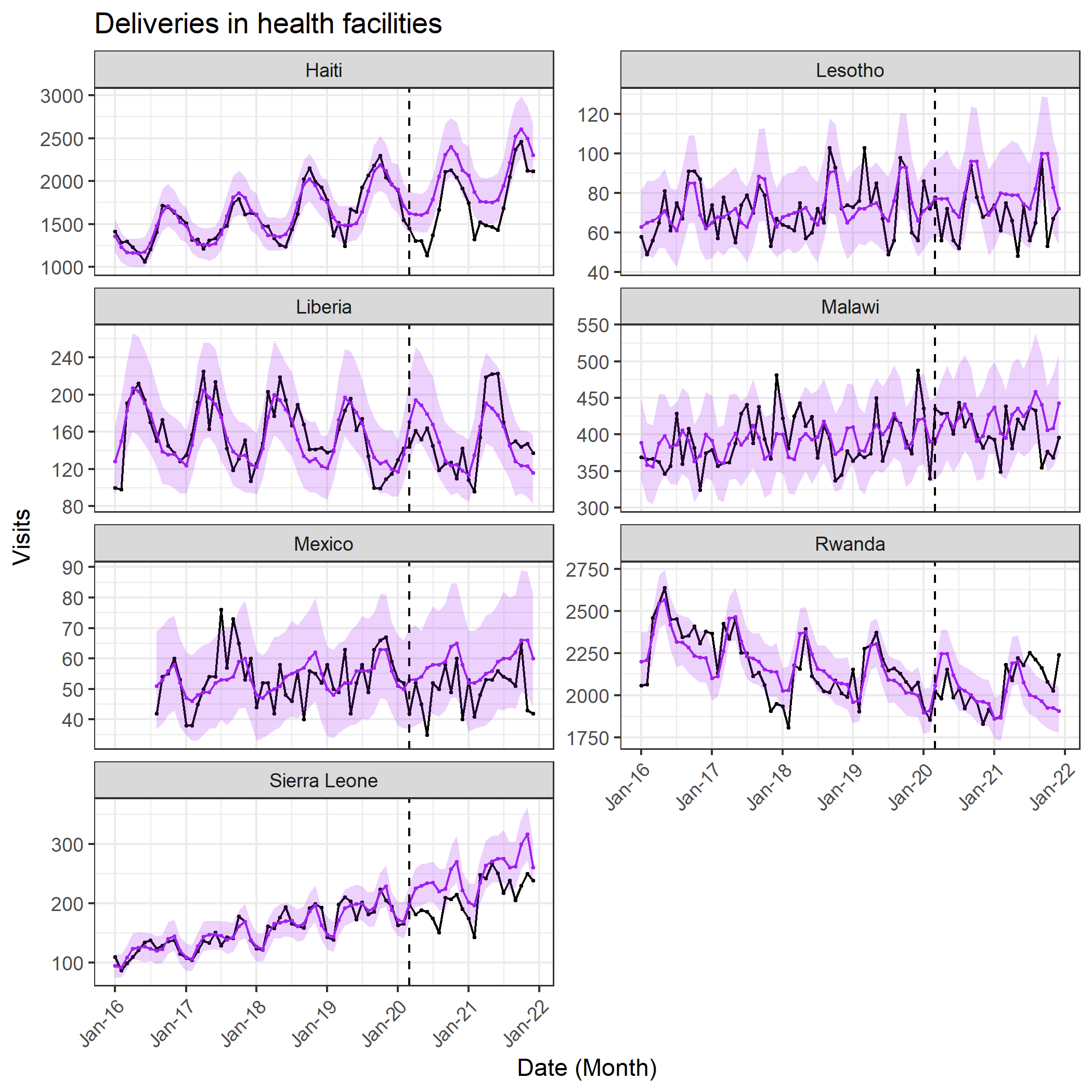

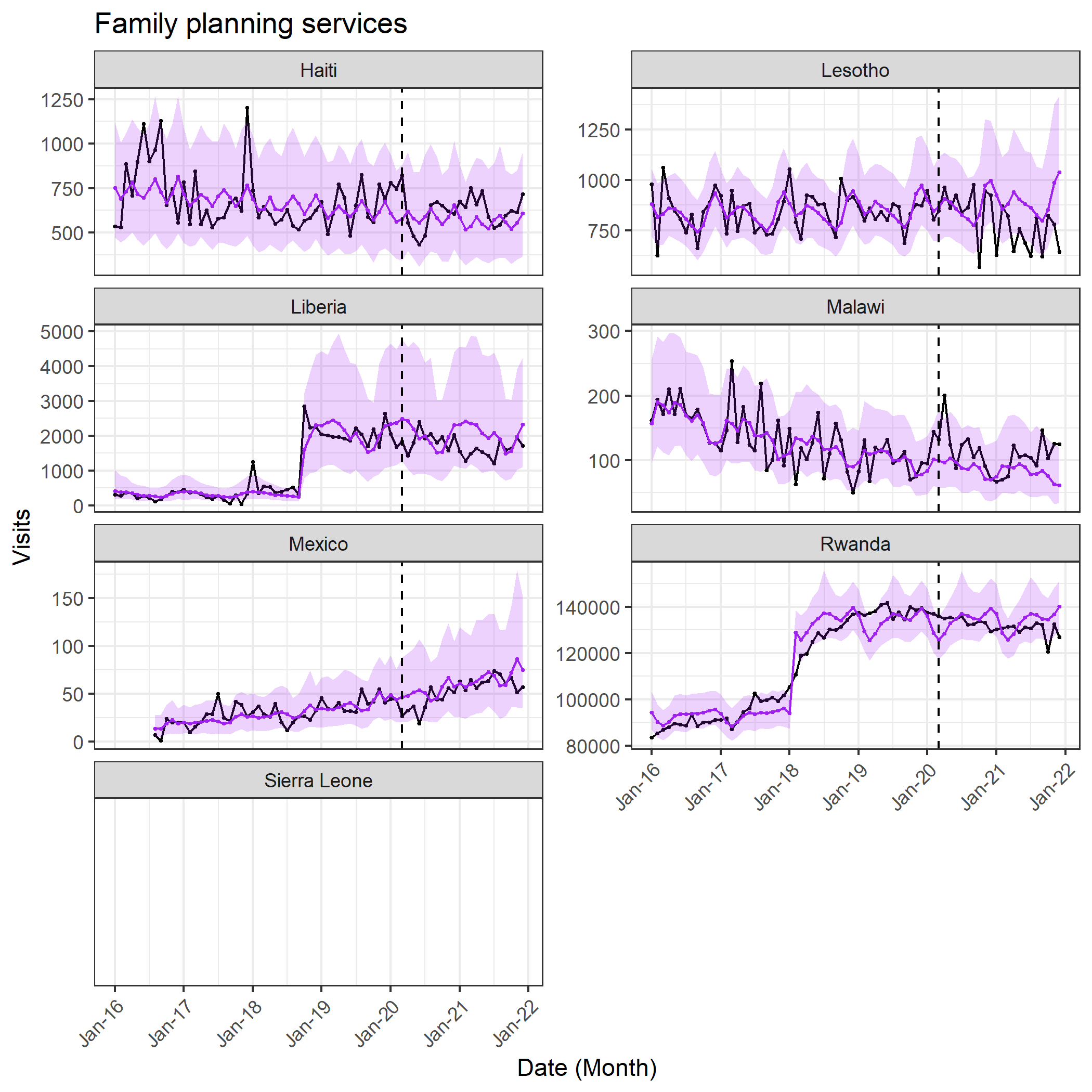


**S3.** Average monthly stringency index by country and month, 2020-2021


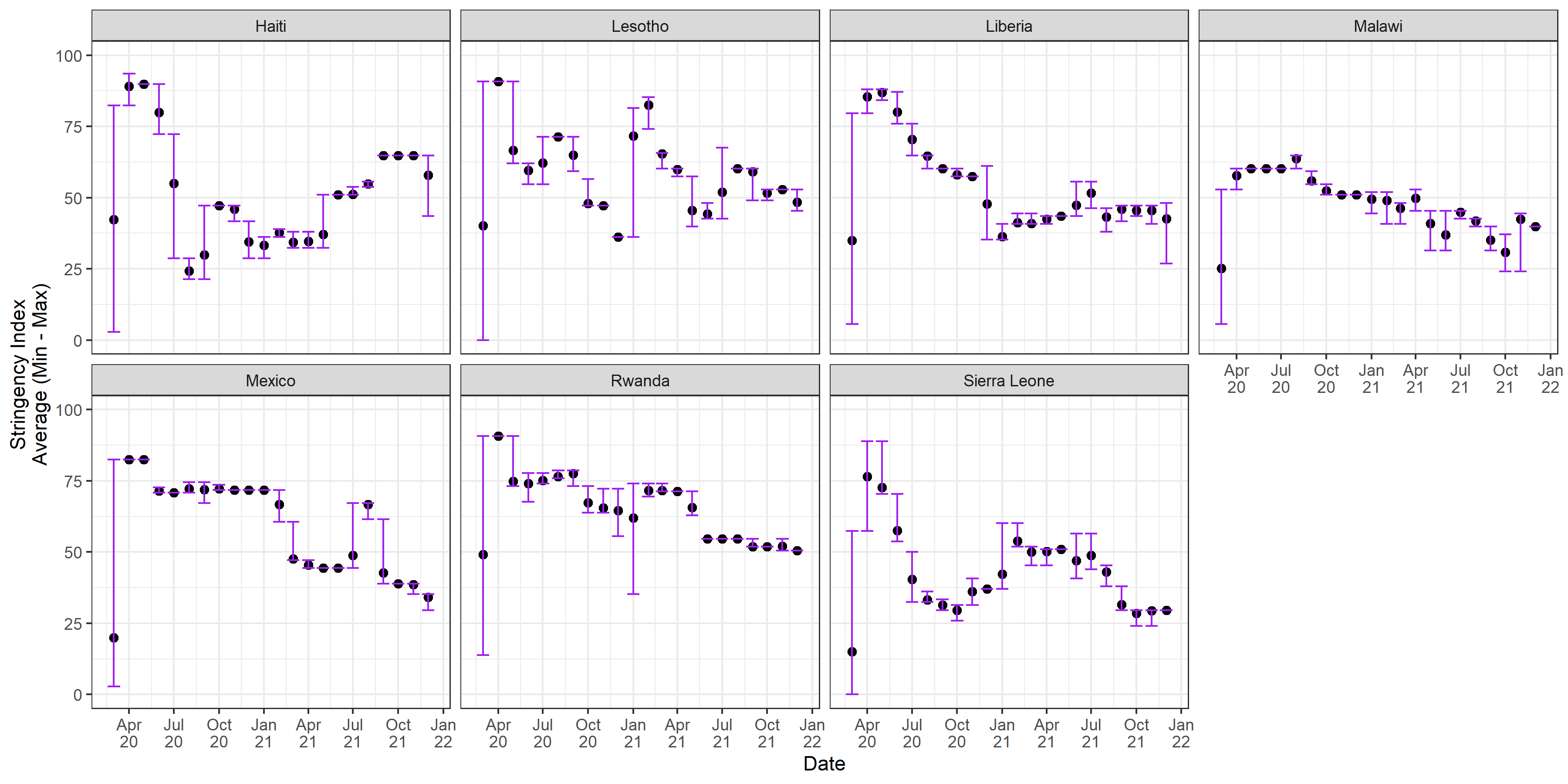


**S4.** Full regression output for the relationship between the average monthly stringency index (fixed effect) and estimated deviation in health service utilization, adjusted for country (random effect) and 1-month lagged monthly COVID-19 cases per 1,000 people (fixed effect)

| Outcome | Regression type | Indicator | Intercept | | Stringency index | | Lagged COVID cases per capita | | Residual |
| --- | --- | --- | --- | --- | --- | --- | --- | --- | --- |
|  |  |  | Beta (95% CI) | p-value | Beta (95% CI) | p-value | Beta (95% CI) | p-value | Standard error |
| Estimated proportion deviation | Linear | Outpatient visits | -0.0187 (-0.1918, 0.1565) | 0.8343 | -0.0039 (-0.0051, -0.0016) | 0.0002* | 0.0426 (-0.0062, 0.0917) | 0.0915 | 0.1480 |
|  |  | Deliveries in health facilities | -0.0308 (-0.1190, 0.0581) | 0.5043 | -0.0011 (-0.0023, 0.0004) | 0.0578 | 0.0170 (-0.0150, 0.0482) | 0.2938 | 0.1076 |
|  |  | Family planning services | -0.0096 (-0.2242, 0.1998) | 0.9302 | -0.0005 (-0.0028, 0.0026) | 0.9721 | 0.0409 (-0.0276, 0.1060) | 0.2288 | 0.2238 |
| Binary indicator for a significant decrease in utilization (below 95% PI) | Logistic | Outpatient visits | -1.4266 (-4.1309, 1.2778) | 0.3012 | 0.0472 (0.0137, 0.0807) | 0.0057* | -0.9162 (-2.0205, 0.1881) | 0.1039 | NA |
|  |  | Deliveries in health facilities | -1.2373 (-2.9797, 0.5052) | 0.1640 | -0.0012 (-0.0275, 0.0251) | 0.9288 | -0.0246 (-0.7710, 0.7218) | 0.9485 | NA |
|  |  | Family planning services | -0.8000 (-3.6191, 2.0191) | 0.5781 | -0.0272 (-0.0751, 0.0207) | 0.2652 | -0.9524 (-2.5232, 0.6185) | 0.2347 | NA |

**indicates a p-value below the 0.05 significance-level cutoff*

**S5.** Correlation plot of stringency index components


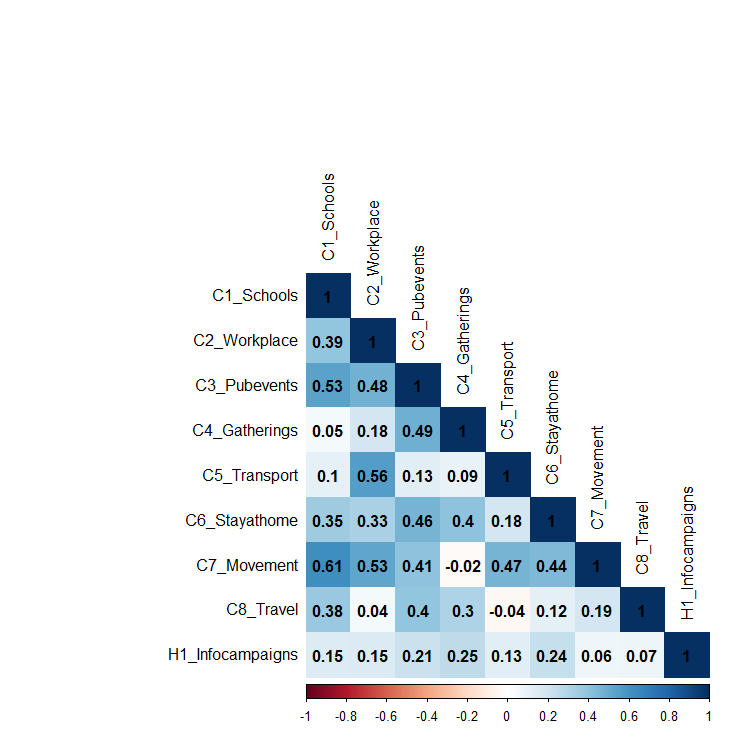


**S6.** Relationship between the components of the average monthly stringency index (fixed effect) and estimated deviation in outpatient visits via linear regression, adjusted for country (random effect) and 1-month lagged monthly COVID-19 cases per 1,000 people (fixed effect)

| Component | Component beta coefficient | p-value |
| --- | --- | --- |
| C1 – School closing | -0.0496 | 0.0003* |
| C2 – Workplace closing | -0.0670 | 0.0015* |
| C3 – Cancel public events | -0.0300 | 0.1882 |
| C4 – Restrictions on gatherings | 0.0044 | 0.7630 |
| C5 – Close public transport | -0.0891 | 0.0056* |
| C6 – Stay at home requirements | -0.0519 | 0.0255* |
| C7 – Restrictions on internal movement | -0.0860 | <0.0001* |
| C8 – International movement controls | -0.0240 | 0.0654 |
| H1 – Public info campaigns | 0.0767 | 0.6216 |

**indicates a p-value below the 0.05 significance-level cutoff*
